# The central GLP-1: implications for food and drug reward

**DOI:** 10.3389/fnins.2013.00181

**Published:** 2013-10-14

**Authors:** Karolina P. Skibicka

**Affiliations:** Department of Physiology, Institute of Neuroscience and Physiology, The Sahlgrenska Academy at the University of GothenburgGothenburg, Sweden

**Keywords:** food reward, ethanol reward, GLP-1, gut peptides, ventral tegmental area, dopamine, exendin 9–39, liraglutide

## Abstract

Glucagon-like-peptide-1 (GLP-1) and its long acting analogs comprise a novel class of type 2 diabetes (T2D) treatment. What makes them unique among other T2D drugs is their concurrent ability to reduce food intake, a great benefit considering the frequent comorbidity of T2D and obesity. The precise neural site of action underlying this beneficial effect is vigorously researched. In accordance with the classical model of food intake control GLP-1 action on feeding has been primarily ascribed to receptor populations in the hypothalamus and the hindbrain. In contrast to this common view, relevant GLP-1 receptor populations are distributed more widely, with a prominent mesolimbic complement emerging. The physiological relevance of the mesolimbic GLP-1 is suggested by the demonstration that similar anorexic effects can be obtained by independent stimulation of the mesolimbic and hypothalamic GLP-1 receptors (GLP-1R). Results reviewed here support the idea that mesolimbic GLP-1R are sufficient to reduce hunger-driven feeding, the hedonic value of food and food-motivation. In parallel, emerging evidence suggests that the range of action of GLP-1 on reward behavior is not limited to food-derived reward but extends to cocaine, amphetamine, and alcohol reward. The new discoveries concerning GLP-1 action on the mesolimbic reward system significantly extend the potential therapeutic range of this drug target.

## Introduction

The alarming rates of obesity in the western world clearly indicate that we have not yet adapted to the dietary challenges engendered by the environment of readily available cheap calories. Inability to limit excessive food intake is likely a key process contributing to uncontrolled weight gain. It is clear that food, especially palatable, calorie-dense, obesogenic food, is rewarding. The high hedonic value and motivational incentive of food are the main culprits for overeating or eating beyond the immediate metabolic need; here referred to as food reward behavior. Thus, in order to develop effective anti-obesity treatments it is of high interest to discover the mechanisms that can limit the hedonically-driven eating and food reward behavior. One potentially promising therapeutic is glucagon-like-peptide 1 (GLP-1). Endogenous GLP-1 is produced in the intestinal L-cells and the hindbrain (Han et al., 1986; Jin et al., 1988; Larsen et al., 1997a; Reimann et al., 2008). GLP-1 receptors (GLP-1R) can also be stimulated exogenously via long lasting GLP-1 analogs (Hayes et al., 2010; Graham et al., 2012; Parkes et al., 2013). Several GLP-1 analogs, among them exendin 4 (EX4; Byetta) or liraglutide (Victoza), are approved for clinical use in type 2 diabetes (T2D) patients to improve glycemic control (Wang et al., 1997; Greig et al., 1999; Agerso et al., 2002; Drucker et al., 2010). Much has been learned about the anatomical, neurochemical, and functional suppressive effects of GLP-1 or its analogs on food intake; GLP-1's ability to suppress food reward behavior is a new concept.

The goal of this review is to extend the understanding of the neural circuitry mediating the intake inhibitory effects of GLP-1 beyond the hypothalamus and the hindbrain and into the mesolimbic areas. Also discussed here will be the behavioral consequences of the aforementioned expansion of the range of GLP-1 impact, into the mesolimbic system. Namely the new behavioral and physiological effects of GLP-1, such as the regulation of food and drug reward.

## GLP-1 anorexia beyond the direct hypothalamus/hindbrain action

Central or peripheral GLP-1 injection reduces food intake in rodents and man (Turton et al., 1996; Larsen et al., 1997b; Naslund et al., 1999; Langhans, 2000; Hayes et al., 2008; Astrup et al., 2009, 2012). To date the literature has primarily focused on hypothalamic and brainstem nuclei as the key central nervous system (CNS) targets for anorexic and to some extent glucoregulatory effects of GLP-1 (Shughrue et al., 1996; McMahon and Wellman, 1998; Schick et al., 2003; Hayes et al., 2008, 2009; Sandoval et al., 2008). This topic has already been discussed by a number of excellent reviews (Holst, 2004; Hayes et al., 2010; Trapp and Hisadome, 2011). This hypothalamus/hindbrain focused model of GLP-1 action, however, does not easily accommodate the recent findings showing that GLP-1R stimulation reduces food reward behavior (Dickson et al., 2012). This observation was important as it supports the presumption that the range of impact of GLP-1 on food intake extends beyond homeostatic (or metabolic, need based) food intake. It brings attention to potential activity of GLP-1 in areas classically associated with reward behavior such as the ventral tegmental areas (VTA) and the nucleus accumbens (NAc). The VTA and its dopaminergic projections to the NAc orchestrate goal-directed motivated behavior to obtain natural reinforcements like food or sex (Wise and Bozarth, 1984; Wise, 2004a,b, 2006). It is also increasingly clear that addictive drugs (Koob, 1992) can hijack the reward system, a system originally evolved to motivate the drive for natural rewards. While the reward control brain centers are neuroanatomically separated they are not entirely disconnected from the classic homeostatic centers, and bidirectional communication between both regions takes place under physiological conditions. Many of the classic hormones regulating feeding have a direct impact on the mesolimbic VTA/NAc neurons (Abizaid et al., 2006; Fulton et al., 2006; Hommel et al., 2006; Palmiter, 2007; Abizaid, 2009; Vucetic and Reyes, 2010; Skibicka and Dickson, 2011; Skibicka et al., 2011; DiLeone et al., 2012). GLP-1 is the newest member of this group.

### Mesolimbic reward system GLP-1 receptor expression

Already in 1999 the first neuroanatomical indication for a potential role of GLP-1 in reward emerged. GLP-1R mRNA and GLP-1 immunoreactivity was detected in the VTA and NAc, as well as other reward associated areas including lateral hypothalamus, lateral habenula, hippocampus, and substantia nigra (Merchenthaler et al., 1999). Identification of the peptide and receptors, however, does not by itself substantiate a functional relevance, but evidence for a physiological role of these receptor populations emerged 13 years later and will be discussed here.

### Afferent and efferent innervation of the hindbrain GLP-1-producing neurons

**Figure 1:**
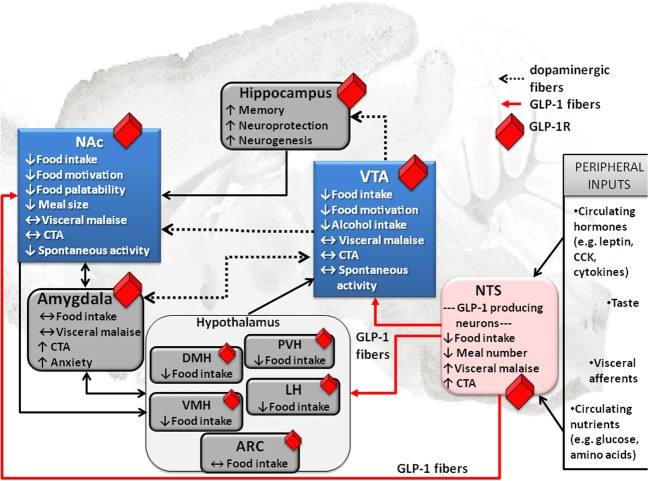
**Effect of GLP-1 on food intake and associated behaviors is neuroanatomicaly distributed**. Local application of GLP-1 or GLP-1 analogs (e.g., EX4) into the VTA or the NAc alters food motivation/reward. Moreover, many other GLP-1R expressing CNS sites that directly respond to GLP-1 have clear connections to the mesolimbic dopamine circuitry, key in food reward behaviors (indicated in blue). This neuroanatomical distribution of GLP-1R potentially allows for a multi-center, wide-spread impact of GLP-1on food reward behavior, at several levels of the CNS. Furthermore, it appears that GLP-1 influences feeding in different brain regions by partly overlapping and partly distinct mechanisms. Several GLP-1 terminal sites have been confirmed (in red). Notably two of them are mesolimbic; VTA and NAc. Presumably, however, most GLP-1R expressing sites would receive their GLP-1 supply from the only source of GLP-1 in the brain, the hindbrain NTS GLP-1-producing neurons. This neuroanatomical architecture places the GLP-1 system as a central sensor of an array of key circulating factors and neural inputs from the viscera and tongue that is immediately able to integrate and relay the information to the mesolimbic centers. Prefrontal cortex, PFC; nucleus tractus solitarius, NTS; ventral tegmental area, VTA; paraventricular nucleus of the hypothalamus, PVH; lateral hypothalamus, LH; arcuate nucleus of the hypothalamus, ARC; ventromedial nucleus of the hypothalamus, VMH; dorsomedial nucleus of the hypothalamus, DMH; conditioned taste aversion, CTA.

Unlike many of its gut or fat hormone counterparts (ghrelin or leptin) for which there is little evidence of central production, GLP-1 can be made in the brain. At the level of the CNS GLP-1 is produced in the hindbrain, primarily in the nucleus of the solitary tract (NTS) (Han et al., 1986; Jin et al., 1988; Larsen et al., 1997a; Reimann et al., 2008). This is a very strategic location for the GLP-1 neurons because of the impressive range of energy balance relevant inputs received by the NTS (Figure 1). NTS receives vagal input from the gastrointestinal tract and gustatory input from the tongue (Grill and Kaplan, 2002; Grill and Hayes, 2009, 2012). Furthermore, the NTS located GLP-1 neurons extend their dendrites into the circumventricular area postrema, an area with fenestrated capillaries that can sample blood borne factors (Llewellyn-Smith et al., 2011). Also projections of these hindbrain GLP-1 neurons are widely distributed, giving neuroanatomical support for the diverse physiological and behavioral effects of the endogenous GLP-1. Key mesolimbic reward areas, like the VTA and the NAc, are innervated by the GLP-1 producing neurons, potentially allowing hindbrain GLP-1 to modulate modulate reward behavior directly. Ascending fibers from the caudal NTS, identified with anterograde tracing, terminate in the VTA and the NAc (Rinaman, 2010). In fact, nearly one third of all the NTS GLP-1-producing neurons send ascending fibers to the VTA and the NAc (core and shell regions) (Dossat et al., 2011; Alhadeff et al., 2012). Thus, GLP-1 neurons are placed in a very influential position, enabling them to sample the hormonal milieu, visceral sensory and gustatory input, integrate and carry this information directly to the mesolimbic system without any intermediate stops.

### GLP-1R-driven activation of the mesolimbic neurons

On the basis of immediate early gene expression analysis it seems that GLP-1 or its analogs can increase neuronal activity in the mesolimbic system. Local application of GLP-1 to the core of the NAc increases the number of neurons expressing the immediate early gene, c-fos, in this region (Dossat et al., 2011). Similar activation can even be obtained with a peripheral GLP-1 analog, EX4, injection (Labouesse et al., 2012). Given that EX4 is administered peripherally as a part of the anti-diabetic treatment regimen, data linking peripheral GLP-1 analog injections to altered neuronal activity in reward areas may be relevant in the clinical and therapeutic setting.

### Mesolimbic GLP-1R driven food-oriented behaviors

Even though the research on the physiological role of the mesolimbic GLP-1 activation is in its early stages the first studies addressing this question provide compelling evidence for an important role of the GLP-1 system in the mesolimbic circuitry and resulting reduction in food intake. Based on the earlier discussed receptor expression, innervation and their crucial role in behavioral control two mesolimbic nuclei, the VTA and the NAc, became the first targets of studies exploring behavioral consequences of the mesolimbic GLP-1R activation. The VTA is a key nucleus that modulates reward behavior and that harbors the cell bodies of dopamine neurons (Wise and Bozarth, 1984; Koob, 1992). Selective and local VTA microinjection of EX4 consistently yields reduction in food intake and body weight. These intake suppressive effects are not macronutrient specific since the intake of both palatable (high-fat or high-sugar) food and normal chow (Alhadeff et al., 2012; Dickson et al., 2012) is reduced by GLP-1R activation. The finding that exogenous stimulation of GLP-1R results in suppression of food intake irrespective of its macronutrient content is perhaps consistent with previous data that indicate that all macronutrients (carbohydrates, fat, proteins) can induce the release of GLP-1 from the intestinal L-cells (Reimann, 2010; Diakogiannaki et al., 2012). Intake of chow, however, is only reduced if the rats are overnight fasted or, in *ad libitum* fed rats, if the chow is available as the only source of calories (Dickson et al., 2012). In contrast, if a choice between chow and high-fat diet is given to satiated rats EX4 appears to selectively reduce the high-fat intake but surprisingly increase the chow intake (Alhadeff et al., 2012). These findings can lead us to conclude that VTA GLP-1R activation might result in a lack of preference for high-energy/fat food. Similar results were reported for the selective stimulation of the GLP-1R in the NAc (Dossat et al., 2011; Alhadeff et al., 2012; Dickson et al., 2012). NAc is a terminal site for the dopaminergic projections originating in the VTA; it can also directly communicate with the lateral hypothalamus, an important interface between the homeostatic and reward circuits. It can be divided into two subregions, the shell and the core; contributions of these two subregions to the control of motivated reward behavior might differ (Di Chiara, 2002). GLP-1 projections are neuroanatomically positioned to influence both subregions, since GLP-1R and innervating fibers can be found in both the shell and the core. Like the VTA GLP-1R, those in the NAc contribute to the intake suppressive responses by GLP-1. Localized delivery of EX4 to the shell or core region reduces high-fat or sucrose intake and body weight. However, in NAc higher doses of EX4 are required to reduce chow intake in both fasted and *ad libitum* fed rats compared with those effective in the VTA, indicating perhaps that NAc is somewhat less sensitive to the intake reducing effects of EX4 (Alhadeff et al., 2012; Dickson et al., 2012). The intake suppressive effect is not unique to the GLP-1 analogs as the native GLP-1 peptide can similarly reduce chow intake when locally delivered to the NAc core (but not shell) (Dossat et al., 2011). Noteworthy, the reported reductions in food consumption appear to be associated with a reduction in body weight (Alhadeff et al., 2012; Dickson et al., 2012). This is an important observation as it may imply that targeting the mesolimbic reward system with GLP-1 agonists may be a viable weight loss strategy.

Together, these data support a role for mesolimbic GLP-1Rs in the regulation of food intake, irrespective of the macronutrient composition of the food. These results are important from a clinical perspective as a reduction in food intake accompanied by weight loss is a desirable outcome in an obese patient. They do not, however, address the question of the physiological role of the endogenously released GLP-1 in these mesolimbic areas. This question has been pursued through focal microinjections of a GLP-1R antagonist into the mesolimbic nuclei. Utilizing this methodology, several reports provide convincing evidence that endogenous GLP-1 released in the VTA and the NAc is in fact necessary for food intake control and blockade of its signal can lead to increased food intake (Dossat et al., 2011; Alhadeff et al., 2012; Dossat et al., 2013). Further support for this idea is provided by Dossat et al. (2013) who report that tonic release of GLP-1 in the NAc core is necessary to reduce the palatability of a sucrose solution and also participates in limiting the size of the sucrose meal. Supportively, both the size of a sucrose meal in rats and also the rate of licking during the first meal, an effect often indicative of a perceived increase in palatability, were increased by local microinjection of a GLP-1R antagonist in the NAc core (Dossat et al., 2013). Interestingly, the recently reported NAc manipulation did not alter non-caloric sweet saccharine consumption (Dossat et al., 2013), suggesting that GLP-1 released in the NAc likely interacts with calories but not taste of the sugary solution. It is worth noting that bypassing the taste signaling by infusing glucose directly into the stomach is sufficient for evoking the rewarding properties of glucose and the associated dopamine release in the NAc (Ren et al., 2010). Thus, it is plausible that the hindbrain GLP-1 neurons are a part of the ascending pathway activated by intragastric glucose, with a role to limit reward responses perhaps.

In the previous paragraphs we have reviewed evidence for the clear suppression of food intake by mesolimbic GLP-1 activation. What is left unresolved is the mechanism(s) responsible for this reduction. Previously discussed data hint at a potential reduction in palatability evoked from the core of the NAc, understood as less pleasure obtained from food and hence leading to less food consumption. The mesolimbic system is, however, key to regulating the incentive salience or motivation (Berridge, 1996). Thus, in the next section the available data on a potential role of the GLP-1 system in motivation will be discussed.

Reviewing the literature it can be somewhat difficult to obtain a consensus on a definition of the now frequently used term, “food reward.” As mentioned earlier, for the purposes of this review this term will refer to eating beyond the immediate caloric need, eating for the hedonic value of food, or heightened incentive salience or motivation to eat. Thus, reducing the motivation to eat is one way to reduce food reward. Food motivation can be measured in animal models (and recently also in human subjects) with an operant procedure for food in which earning each food pellet requires more work than the one before, reflecting craving and wanting for food; this test is called progressive ratio operant procedure (Hodos, 1961; Miras et al., 2012). It is a plausible mechanism via which GLP-1 may lead to intake suppression since activation of GLP-1 via EX4 injection was shown to decrease the motivation to obtain food (Dickson et al., 2012), in a progressive ratio operant conditioning. This effect was rather striking as the central (ventricular) EX4 injection produced an impressive 80% reduction in rewards earned. The food reward-reducing effect of EX4 is further confirmed in another test of food reward, the conditioned place preference test, in which rats conditioned to spend most of their time in an environment previously paired to chocolate pellets lost that conditioned preference when injected with EX4 (Dickson et al., 2012). Of course, these results obtained with peripheral or central infusions of the agonist, while of some clinical relevance (peripheral route), do not address the issue of the neural substrate underlying these reward effects. This issue has been pursued through combining localized mesolimbic delivery of EX4 with the sucrose-motivated progressive ratio test. The reported results are consistent with the hypothesis that the key mesolimbic areas, the VTA and the NAc, represent the neural substrate driving the food reward suppressing effect of GLP-1R stimulation. Selective intra-VTA or intra-NAc EX4 application reduces the incentive value for sucrose (Dickson et al., 2012). Again, the VTA was more sensitive to GLP-1R stimulation. This VTA GLP-1R driven reduction in sucrose reward behavior was shown in both overnight food restricted and *ad libitum* fed rats indicating that this response is robust (Dickson et al., 2012). When sucrose-driven operant behavior is performed in food-restricted rats, the motivation to work for sugar is generally reliable and high for all rats. This is in contrast to the *ad libitum* condition in which some rats still choose to expend a significant amount of work for their sugar reward (high-responders); others, however, expend only a fraction of the work they are willing to do in a restricted state (low-responders) (Dickson et al., 2012). This interesting observation was used to show that these innate differences in *ad libitum* fed rats may interact with GLP-1R-driven reward responses. This is reflected by data showing that EX4 is mostly effective in high-responders, leaving the responses of low-responders intact.

The classic mesolimbic reward areas are clearly important in driving the effect of GLP-1 on food reward; GLP-1R, however, are detected in several other nuclei that contribute to the different aspects of reward control. Substantia nigra is one such interesting candidate area. The crucial role of the substantia nigra dopaminergic neurons in operant conditioning for food emerged from data showing that selective restoration of dopamine production in substantia nigra or its primary terminal target, the dorsal striatum, is sufficient to restore the motivation to work for food in mice that lack dopamine otherwise (Sotak et al., 2005; Robinson et al., 2006, 2007; Palmiter, 2008). GLP-1R has been detected in the substantia nigra (Merchenthaler et al., 1999), its role in food reward behavior is unexplored, but is certainly worth considering in future studies. Interestingly, while we do not know the role of GLP-1 in this circuit on food reward several studies have explored the therapeutic potential of nigro-striatal GLP-1 action in animal models of Parkinson's disease (Harkavyi et al., 2008; Kim et al., 2009; Abuirmeileh et al., 2012; Holscher, 2012) with great success culminating now with clinical trials.

### Competing and confounding behaviors

Certain limitations should always be considered when a suppression of ingestive or motivated behavior is obtained. In the case of GLP-1 two most prominent limitations to drawing specific conclusions about the role of the peptide in regulation of reward are (1) nausea or visceral illness and (2) the non-specific motor disturbance.

#### Nausea/aversion

The concern with nausea resulting from GLP-1 should not come as a surprise as this is in fact the most common side effect reported in patients receiving GLP-1 analog treatment (Calara et al., 2005). This clinical observation is closely mimicked in preclinical rodent studies, in which signs of visceral illness, nausea or conditioned taste aversion after EX4 or GLP-1 treatment have been clearly demonstrated (Thiele et al., 1997, 1998; Rinaman, 1999a,b; Seeley et al., 2000; Kinzig et al., 2002; Kanoski et al., 2012). It seems, however, that while stimulation of some GLP-1R expressing neuronal populations is responsible for reducing food intake in association with nausea or aversion stimulation of others can lead to food intake reduction via mechanisms independent of nausea (McMahon and Wellman, 1998; Alhadeff et al., 2012; Dickson et al., 2012; Kanoski et al., 2012). It is, of course, warranted to consider this possibility carefully as the mesolimbic system underlies the establishment of aversion responses. Three reports to date suggest that VTA and NAc GLP-1Rs induced suppression of food intake and reward is not associated with visceral illness (Dossat et al., 2011; Alhadeff et al., 2012; Dickson et al., 2012). The fact that a similar conclusion, the lack of association between nausea and GLP-1 in reward areas, was obtained across different laboratories, diverse methods of visceral illness assessment and different GLP-1R agonists makes this a much more compelling argument. Relevant studies were conducted in rodents in which conditioned taste aversion and kaolin consumption were used to evaluate potential nausea or malaise associated with EX4 or GLP-1. Kaolin consumption is a form of PICA response (consumption of non-nutritive substances) that is indicative of visceral malaise (Takeda et al., 1993; De Jonghe et al., 2009). The PICA response has been used extensively to study visceral malaise in rats, a species that cannot vomit. VTA and NAc shell directed EX4 application, at doses that clearly reduce food intake and reward behavior, does not induce consumption of kaolin. Similarly intra-NAc core GLP-1 administration at a dose that reduced chow intake did not elicit a conditioned taste aversion to saccharine, indicating that the rats did not find the accumbal GLP-1R stimulation aversive (Dossat et al., 2011). The collective value of these findings is not only in offering support to the hypothesis that mesolimbic GLP-1 has a specific role in reward behavior but also in emphasizing the neuroanatomical separation of the reward and visceral illness mechanisms of GLP-1. Currently this information is of little benefit to the patients receiving GLP-1 based therapy that is applied peripherally and provides drug access presumably to all brain GLP-1R populations simultaneously. However, the fact that the potentially clinically beneficial food reward suppressing effects of GLP-1R stimulation can be disassociated from the clinically undesirable nausea offers promise for a future therapy selectively targeting the mesolimbic reward system GLP-1R populations that could be free of this most common adverse effect of GLP-1 analog therapy.

#### Motor disturbances/hypoactivity

Another concern with therapies targeting the mesolimbic system is that the behavioral outcomes obtained are not specific to ingestive behavior and mediated via a general increase or decrease in physical activity. This becomes a valid concern for GLP-1 as some studies indicate that peripheral GLP-1R agonist application can result in a reduction in spontaneous motor activity (Mack et al., 2006; Erreger et al., 2012). Other reports, however, fail to find this GLP-1/EX4 associated hypoactivity in otherwise normal rats or mice (Talsania et al., 2005; Hayes et al., 2008). Local, intra-VTA GLP-1R activation appears to align with the latter reports and does not result in any changes in non-goal oriented motor activity (Dickson et al., 2012). This is in contrast to the NAc GLP-1R stimulation which led to a brief (10 minute long) reduction in activity, during the 1 h long testing period. Considering how short-lived the hypoactivity period was, it seems unlikely that it is a major contributing factor to reward-associated behavioral effects of EX4 that lasted for a period of several hours. However, this possibility could not be entirely eliminated based on the available data. Nevertheless, the results obtained with VTA GLP-1R population suggest that the suppression of food reward and intake can be fully disassociated from any changes in general motor activity. It is worth mentioning that in the literature evaluating the rewarding effects of psychostimulants a change in physical activity is not considered problematic, as many psychostimulants like cocaine or amphetamine are associated with hyperactivity, and this behavior seems to be intimately linked to increases in dopamine (Imperato and Di Chiara, 1986; Wise and Bozarth, 1987). Thus, a reduction of hyperactivity is often translated to mean a reduction in NAc dopamine release and used as a proxy measure for a reduction of the rewarding/addictive properties of a given psychostimulant. If this line of thinking is applied to the mesolimbic GLP-1R activation, it is plausible that while NAc GLP-1R activation is associated with a suppression of the dopamine signal, the VTA GLP-1R reduce reward behavior via another mechanism. This is, however, rather speculative and requires future investigation that should involve a direct measurement of the dopamine release after intra-VTA or intra-NAc GLP-1 application during a food reward task *in vivo*.

## GLP-1 impact on alcohol intake and reward

Sugars are clearly rewarding; when sugars undergo alcoholic fermentation alcohol is one of the resulting products. While alcohol is reinforcing in itself (Henningfield and Meisch, 1975), it is the only addictive substance that also provides a source of calories. A number of studies highlight the importance of the gut-brain signaling in the regulation of alcohol intake (Crespi, 1998; Leggio, 2010). Thus, it is not surprising that the initial discovery of the impact of GLP-1 on food reward opened up the question whether GLP-1R stimulation could be beneficial for curbing alcohol intake. The link between alcohol and GLP-1 starts already in the gut, as alcohol intake can result in an elevated level of gut-produced GLP-1 in rats (Davis et al., 2012b) suggesting a possible relationship between alcohol intake and GLP-1. Direct evidence for this relationship followed this initial discovery and included findings showing that peripheral injections of GLP-1 or the GLP-1 analog, EX4, reduce alcohol consumption in rodents (Davis et al., 2012b; Shirazi et al., 2013). Interestingly, in both studies this ability to reduce alcohol consumption seems to be dependent on the baseline consumption levels. The consumption of alcohol is significantly reduced, but only in genetically-determined alcohol-preferring rats or those selected from an outbred population for high-alcohol consumption. In contrast, low-alcohol consuming rats show little change in their alcohol drinking after GLP-1R stimulation. The suppressing effect of GLP-1 on alcohol intake is linked to suppressed alcohol reward, an idea now tested in both rats and mice with two different tests of alcohol reward behavior, adding strength to the conclusion. Mice treated with GLP-1 do not show a conditioned place preference to alcohol (Shirazi et al., 2013) and alcohol-preferring rats injected with EX4 reduce their operant responding for an alcohol solution (Davis et al., 2012a). Peripheral injection of EX4 also appears to reduce alcohol-induced accumbal dopamine release, findings consistent with the idea that a peripheral EX4 treatment can have an impact on the mesolimbic dopamine system (Egecioglu et al., 2013). Together these data indicate that the stimulation of GLP-1R is sufficient to reduce alcohol consumption; however, they do not speak to the ability of the endogenous GLP-1 to regulate alcohol intake. To this end, we recently reported that blockade of GLP-1R via a peripheral injection of a GLP-1R antagonist in outbred Wistar rats results in increased alcohol consumption (Shirazi et al., 2013). Thus, endogenous GLP-1 makes a significant contribution to the normal regulation of alcohol intake and when that endogenous signal is taken away the rats drink more alcohol. In contrast, the same GLP-1R blockade was not sufficient to elevate alcohol drinking in alcohol-preferring rats, perhaps reflecting a differential alcohol intake regulation in this strain (Davis et al., 2012b). Importantly, the effect of GLP-1 on alcohol intake may be driven locally by mesolimbic VTA GLP-1R activation, since acute intra-VTA injection of GLP-1 or EX4 reduces overnight alcohol consumption by nearly 30%, (Shirazi et al., 2013). This newly emerging alcohol-suppressing effect of GLP-1 is certainly of clinical interest. Since GLP-1 analogs are already approved for clinical use in T2D and deemed clinically safe, it is surely an attractive possibility to consider their use for alcohol disorders. It is important to note, however, that the data summarized here include only preclinical studies; thus, future studies are necessary to determine whether this newly discovered effect can be generalized to a clinical population. Furthermore, all discussed reports evaluate only acute effects of the treatment, and evaluation of long-term effects of the GLP-1R stimulation on the alcohol intake has not yet been reported. Nevertheless, collectively these findings provide further support for a pleiotropic impact of GLP-1 on the mesolimbic circuitry and reward behavior.

## GLP-1 impact on psychostimulant and nicotine reward

Considering that the mesolimbic reward system is a key target not only for food and alcohol, but also for psychostimulants and nicotine (Koob, 1992), it is perhaps not surprising that recent work has explored the potential of GLP-1 analogs to alter psychostimulant reward. It must be noted that the effects of a substance to reduce food reward cannot be simply extrapolated to suggest a psychostimulant reward-reducing effect. Melanocortins, for example, are one of the most potent anorexic neuropeptides (Cone, 2005), they reduce food reward behavior (Davis et al., 2011) but may increase mesolimbic dopamine release and psychostimulant reward (Lindblom et al., 2001; Hsu et al., 2005). Thus, a careful and direct evaluation of the role of GLP-1 on the psychostimulant reward was necessary and proved fruitful, since an impact of GLP-1 on both cocaine and amphetamine behavioral response was revealed (Erreger et al., 2012; Graham et al., 2012). Peripheral delivery of EX4 attenuated cocaine reward behavior in mice in a cocaine-induced conditioned place preference test. Perhaps surprisingly, this attenuation of the conditioned preference response was not associated with a reduction in cocaine-induced hyperactivity, even though the doses used were well into the range of those previously shown to induce hypoactivity and 10 to 100-fold higher than those needed for an anorexic response. Since it is often suggested that the cocaine-induced hyperactivity might be a correlate of dopamine release in the NAc, the lack of an effect of EX4 on cocaine hyperactivity might indicate that the reduction in reward value of cocaine occurs via a mechanism outside of or downstream of the dopamine release. In contrast, a peripheral injection of EX4 in rats reduced both basal locomotor activity and amphetamine-induced hyperactivity, which may be indicative of a reduced dopamine signal. If the results of these two studies were to be integrated it could be concluded that the impact of GLP-1R activation on psychostimulant-induced hyperactivity, and by extension dopamine release, may be species (mice vs. rats) or drug (cocaine vs. amphetamine) dependent. Interestingly, peripheral injection of liraglutide was shown to reduce behavior induced by apomorphine (a non-selective dopamine agonist) (Dixit et al., 2013). The effect of liraglutide was strikingly comparable to that achieved by a well-established antipsychotic medication, haloperidol (Dixit et al., 2013). While these are very promising results, it is clear that future studies are needed to determine the mechanisms (intracellular signals and downstream neurotransmitters) behind the impact of EX4 on the specific psychostimulant reward. Furthermore, nothing is known about the contribution of the endogenous GLP-1 to the psychostimulant reward; it would certainly be worthwhile to explore whether changes/dysfunction in the endogenous GLP-1 system are a contributing factor to psychostimulant reward and addiction. This essential contribution of endogenously produced GLP-1 to drug reward is, however, suggested by Tuesta et al. (2012). They show that nicotine reward, tested via a conditioned place preference in mice lacking the GLP-1R, was significantly enhanced (Tuesta et al., 2012). Moreover, the GLP-1R knockout mice self-administered more nicotine than their wildtype counterparts (Tuesta et al., 2012); which could suggest an enhanced motivational salience of nicotine in these mice. These compelling data suggest that the endogenous GLP-1 is necessary for curbing the reward experience from drugs.

## Contribution of GLP-1 to changes in reward behavior after bariatric surgery

Bariatric surgery results in an impressive weight loss, reduced food intake and a lack of compensatory energy expenditure reduction (le Roux and Bloom, 2005; Sjostrom et al., 2007; Buchwald and Oien, 2009; Buchwald, 2010; Carlsson et al., 2012). Some studies also report reduced food reward post-surgery (Shin and Berthoud, 2011; Miras et al., 2012) but this reduction is not detected by others (Mathes et al., 2012). The effect of bariatric surgery on the GLP-1 system is also well-established (Cummings, 2009). The peripheral GLP-1 system is more responsive to both meals and alcohol after bariatric surgery (Davis et al., 2012b). Some reports also suggest that this surgery can lead to reduced alcohol intake and reward in both humans and rodents (Davis et al., 2012b). Thus, it is likely that an elevated circulating GLP-1 contributes to the reduced food and alcohol reward after bariatric surgery. In fact, pharmacological blockade of GLP-1R that is ineffective in sham rats, restores the chow intake of Roux-en-Y gastric (RYGB) operated rats (Abegg et al., 2013). This essential contribution of GLP-1 was not, however, found in a mouse model of vertical sleeve gastrectomy (Wilson-Pérez et al., 2013). Further studies evaluating the effect of longer-term or central GLP-1R stimulation are still needed to entirely eliminate the requirement of GLP-1 signaling for the food/alcohol suppressing effect of bariatric surgery. It is also noteworthy that one recent report indicates an elevated ethanol intake and reward in diet-induced obese rats after bariatric surgery (Hajnal et al., 2012) that might be associated with higher levels of another gut peptide, ghrelin. This is consistent with reports indicating that a small but significant subpopulation of baratric surgery patients drinks more alcohol after the surgery. The involvement of GLP-1 was not suggested by either of these studies. These surprisingly conflicting results in preclinical bariatric surgery models might be associated with the different rat strains used: lean outbred rats, lean alcohol preferring rats vs. diet-induced obese rats, an idea in need of further experimental support. The question: which preclinical bariatric surgery model is the most relevant for the human condition remains to be answered.

## Future prospects

The data reviewed here paint a clear picture of the potent and robust food and drug reward suppressing action of the GLP-1 system. The GLP-1-reward field is, however, very young and much work remains to be done. The results reviewed here need to be confirmed in a clinical population. There is good reason to expect that similar food/drug reward suppressing effects would be obtained in human subjects since most beneficial effects of GLP-1 reported to date are easily translated from preclinical to clinical studies. Nonetheless, direct evidence is still missing. Furthermore, almost all available evidence reflects the acute effects of GLP-1 manipulation on reward; thus long-term studies are still needed to confirm that the reward-suppressing action can be maintained over time and with repeated GLP-1 analog administration.

The food-reward suppressing effect of GLP-1R stimulation needs to be confirmed in the target preclinical and clinical population—obese subjects. Chronic high-fat or high-sugar food intake is associated with changes in the mesolimbic reward system and reward behavior (Li et al., 2009; Vucetic et al., 2011; Sharma et al., 2013). There seems to be a lack of consensus on the exact impact of this chronic overeating on the reward circuitry. Both enhanced and reduced food reward behavior have been suggested, either as a contributor or a result of obesity (Berthoud et al., 2011). In an attempt to reconcile the disparate findings it has been suggested that the activity of food responsive reward circuitry is high before development of obesity and becomes suppressed with time due to excessive consumption of the rewarding food. Thus, it is clear that an effect of a drug on reward in non-obese animals cannot be simply extrapolated to those that are obese and it will be crucial to determine whether the food reward suppressive effects of GLP-1 and analogs is relevant and beneficial in an obese model. Moreover, while GLP-1 analog therapy is clearly effective in regulating blood glucose of obese patients, the anorexic effects of the acute peripheral injections of GLP-1 or EX4 may be attenuated in rats fed a high-fat diet (Williams et al., 2011). Yet evidence also exists suggesting that the anorexic response to a GLP-1 analog, liraglutide, is in fact enhanced and lasts much longer in high-fat fed rats (Mul et al., 2013). Which of these findings is relevant for food reward regulation by GLP-1 remains to be determined.

Studies exploring the CNS effects of GLP-1 could also greatly benefit from a neuroanatomical dissection of the hindbrain GLP-1-producing neuronal population and its projection targets. Peripheral or ventricular exogenous drug application can likely allow for stimulation of most GLP-1R-expressing cell populations simultaneously. It would be of great interest to determine whether it is possible to stimulate, also in a clinical setting, specific GLP-1 populations. One way this could be achieved would be if different hindbrain GLP-1-producing neurons have divergent projection targets and a different set of inputs. For example, if one population activated by factor X projects specifically to the amygdala but another activated by factor Y to the VTA, it would be possible to achieve more precise and selective VTA GLP-1R activation by simply applying factor Y. Elegant studies of this type have been done for example by Leshan and colleagues to show that only a subpopulation of VTA dopamine neurons projecting specifically to the amygdala but not the NAc is activated by leptin (Leshan et al., 2010). The idea of diverse subpopulations for GLP-1 producing neurons is, at this point, purely speculative and remains to be investigated. Furthermore, understanding the functionality of the GLP-1R may help in development of more specific GLP-1 analogs (Patterson et al., 2013). The neurochemical mediators downstream from the mesolimbic GLP-1R are also largely unexplored. Thus, both the intracellular signals engaged by the GLP-1R expressing cells and the downstream neuropeptides and neurotransmitters released await a detailed analysis.

## Concluding remarks

Behavioral feeding responses are evolutionarily conserved and necessary for survival. The neurocircuitry underlying feeding control is complex and requires the coordinated involvement of many likely redundant and parallel brain circuits. Thus, it is likely that the most efficient way to achieve a reduction in food intake would have to involve a simultaneous impact on a number of CNS regions controlling different aspects of feeding regulation from the cognitive, decision-making, memory, and rewarding aspects to the feeling of satiety and hunger. The evidence reviewed here supports the idea that GLP-1 action on the CNS is distributed with many brain nuclei being engaged simultaneously via central GLP-1 to regulate metabolism, and reward-seeking behaviors.

### Conflict of interest statement

The author declares that the research was conducted in the absence of any commercial or financial relationships that could be construed as a potential conflict of interest.
